# Aspirin and prostate cancer prevention

**DOI:** 10.18632/aging.100747

**Published:** 2015-05-21

**Authors:** Adriana C. Vidal, Stephen J. Freedland

**Affiliations:** Department of Surgery, Cedars-Sinai Samuel Oschin Comprehensive Cancer Institute, Los Angeles, CA 90048, USA

Aspirin's anti-cancer effect was first reported in animals in 1972 [1]. Since then, there has been tremendous interest in aspirin, a relatively non-toxic drug, to prevent cancer. Forty years after the initial publication of aspirin's effects on cancer, a meta-analysis from 51 randomized trials in humans demonstrated that daily aspirin use for ≥5 years significantly reduced cancer mortality, an effect that was independent of aspirin dose [2]. However, the effects of aspirin and other non-aspirin non-steroidal anti-inflammatory drugs (NSAIDs) on cancer incidence and prevention are less clear. For example, while the evidence is stronger for NSAIDs and lower risk of cancers such as melanoma, colorectal, breast and lung, the association with prostate cancer risk is inconclusive [3]. Indeed, a meta-analysis found that in European studies, NSAID use was associated with an increased risk of prostate cancer, whereas in North American studies, NSAID use was associated with lower prostate cancer risk [4].

One explanation for the disparate results is detection bias due to PSA screening given that aspirin and NSAIDs lower PSA. Indeed, in North America, where most men are screened with PSA, lower PSA induced by aspirin and NSAIDs would lead to fewer biopsies and fewer cancers detected. This may explain the lower prostate cancer risk with aspirin and NSAIDs use in North America. In contrast, in Europe, where PSA screening is less common, the effect of artificially lowered PSA is less relevant, and there is no “protective” effect of aspirin and NSAIDs use. Also, as aspirin and NSAIDs are used to treat pain, perhaps men in Europe with undiagnosed prostate cancer had pain related to their cancer necessitating aspirin/NSAID use resulting in reverse causation (i.e. the cancer led to pain necessitating aspirin/NSAID use, not the other way around). This would be moot in North America as aggressive screening results in few men presenting with pain at diagnosis. Given these issues, the true association between anti-inflammatory medications and prostate cancer risk is unclear.

To address this, we tested the association between aspirin and non-aspirin NSAIDs and prostate cancer in REDUCE, a randomized trial of dutasteride for prostate cancer risk reduction among men with an elevated PSA and negative pre-study biopsy. Importantly, all men were required to undergo per-protocol biopsies regardless of PSA levels. Among 6,390 men with an on-study biopsy, use of aspirin and/or NSAIDs was associated with 13% reduced risk of prostate cancer and 20% reduced risk of high-grade prostate cancer [5]. These data are consistent with the hypothesis that anti-inflammatory drugs reduce prostate cancer risk, including high-grade prostate cancer, supporting future clinical trials of anti-inflammatory drugs for prostate cancer prevention.

Although reports from Europe are still controversial, a recent study tried to address the PSA screening bias when examining the association between NSAID use and prostate cancer risk. Data were reviewed from the largest component of the European Randomized Study of Prostate Cancer Screening, the Finnish Prostate Cancer Screening Trial [6]. Men were randomized to a no-screening control arm or a screening arm were patients underwent PSA screenings at four-year intervals and if PSA was ≥4ng/ml they received a prostate biopsy. Among 78,615 men, current, but not previous NSAIDs use was associated with increased risk of prostate cancer. The authors concluded that as previous use was unrelated to prostate cancer and current use was linked with increased risk that this suggests the increase risk may result from conditions indicating NSAID usage, like symptoms of undiagnosed prostate cancer. Importantly, whether men underwent PSA screening or not, no evidence of reduced prostate cancer risk was seen.

A limitation of most epidemiological studies is the lack of information on indications for NSAID use, making it difficult to analyze the reasons behind their use. It is known that NSAIDs are used for various conditions including chronic inflammation and coronary artery disease, which may be risks factors for prostate carcinogenesis, thereby confounding the biological effect of aspirin and NSAIDs on prostate cancer risk.

Given the conflicting data and potential harms (GI bleeding), patients should not be prescribed NSAIDs or aspirin for prostate cancer prevention. Instead, we need data from randomized clinical trials. Indeed such a trial was completed in high-risk people for colon cancer, wherein daily aspirin for at least 2 years reduced the risk or colorectal cancer by nearly 60 percent [7].

Finally, a large trial (ADASPIRIN, http://www.addaspirintrial.org/) is underway random-izing patients with colorectal, breast, gastro-esophageal and prostate cancers who all had primary treatment with curative intent to placebo or aspirin. Until the results of this and other much needed trials are complete, we must conclude that we are not quite there yet in terms of NSAIDs/aspirin for prostate cancer prevention.
